# [6-(3,5-Dimethyl-1*H*-pyrazol-1-yl)picolinato](pyridine-2,6-dicarboxyl­ato)copper(II) dihydrate

**DOI:** 10.1107/S1600536808013731

**Published:** 2008-05-14

**Authors:** Fei-Long Hu, Xian-Hong Yin, Kai Zhao, Yu Feng, Cui-Wu Lin

**Affiliations:** a College of Chemistry and Ecological Engineering, Guangxi University for Nationalities, Nanning 530006, People’s Republic of China; b College of Chemistry and Chemical Engineering, Guangxi University, Nanning 530004, People’s Republic of China

## Abstract

In the title complex, [Cu(C_7_H_4_NO_4_)(C_11_H_10_N_3_O_2_)]·2H_2_O, the Cu^II^ atom is in a distorted octa­hedral geometry. The equatorial plane is formed by two N atoms and one O atom from 6-(3,5-dimethyl-1*H*-pyrazol-1-yl)picolinate and by one N atom from pyridine-2,6-dicarboxyl­ate (pdc). Two pdc O atoms occupy the axial positions. Water mol­ecules are hydrogen bonded to the complex mol­ecules, forming a two-dimensional sheet structure.

## Related literature

For the isostructural Ni derivative of the title compound, see: Feng *et al.* (2008 ▶). For other related literature, see: Yin *et al.* (2007 ▶); Zhao *et al.* (2007 ▶).
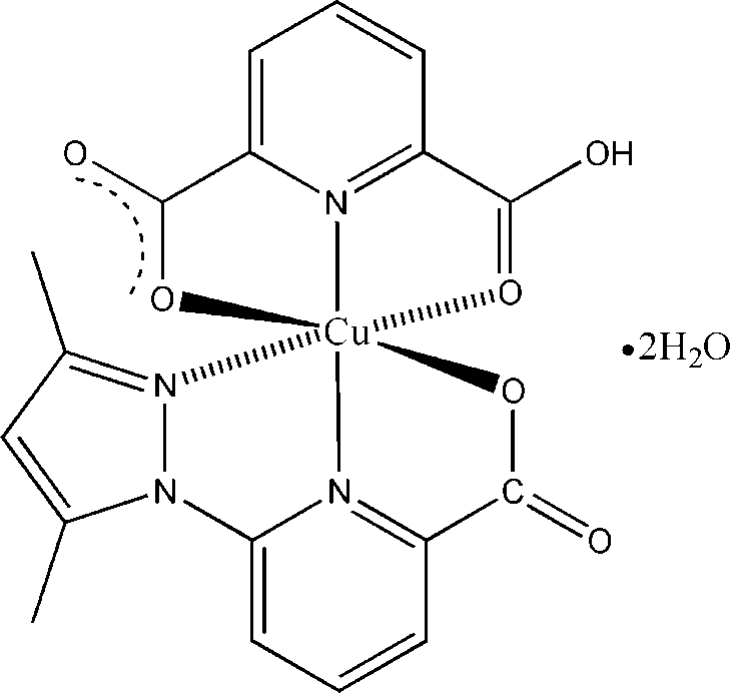


## Experimental

### 

#### Crystal data

[Cu(C_7_H_4_NO_4_)(C_11_H_10_N_3_O_2_)]·2H_2_O
*M* *_r_* = 481.90
Triclinic, 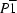

*a* = 9.0040 (10) Å
*b* = 9.0360 (10) Å
*c* = 12.6760 (15) Å
α = 103.932 (3)°
β = 90.289 (2)°
γ = 104.177 (3)°
*V* = 968.25 (19) Å^3^
*Z* = 2
Mo *K*α radiation
μ = 1.19 mm^−1^
*T* = 298 (2) K
0.39 × 0.35 × 0.32 mm

#### Data collection

Bruker SMART CCD area-detector diffractometer
Absorption correction: multi-scan (*SADABS*; Sheldrick, 1996 ▶) *T*_min_ = 0.655, *T*_max_ = 0.684
5068 measured reflections
3361 independent reflections
2844 reflections with *I* > 2σ(*I*)
*R*_int_ = 0.014

#### Refinement

*R*[*F* ^2^ > 2σ(*F* ^2^)] = 0.043
*wR*(*F* ^2^) = 0.113
*S* = 1.03
3361 reflections
280 parameters
H-atom parameters constrained
Δρ_max_ = 0.39 e Å^−3^
Δρ_min_ = −0.30 e Å^−3^

### 

Data collection: *SMART* (Siemens, 1996 ▶); cell refinement: *SAINT* (Siemens, 1996 ▶); data reduction: *SAINT*; program(s) used to solve structure: *SHELXS97* (Sheldrick, 2008 ▶); program(s) used to refine structure: *SHELXL97* (Sheldrick, 2008 ▶); molecular graphics: *SHELXTL* (Sheldrick, 2008 ▶); software used to prepare material for publication: *SHELXTL*.

## Supplementary Material

Crystal structure: contains datablocks I, global. DOI: 10.1107/S1600536808013731/zl2096sup1.cif

Structure factors: contains datablocks I. DOI: 10.1107/S1600536808013731/zl2096Isup2.hkl

Additional supplementary materials:  crystallographic information; 3D view; checkCIF report

## Figures and Tables

**Table 1:** Hydrogen-bond geometry (Å, °)

*D*—H⋯*A*	*D*—H	H⋯*A*	*D*⋯*A*	*D*—H⋯*A*
O4—H4⋯O7^i^	0.82	1.69	2.477 (4)	159
O7—H7*D*⋯O8	0.85	1.89	2.637 (4)	145
O7—H7*E*⋯O6	0.85	1.73	2.524 (4)	154
O8—H8*A*⋯O5	0.85	2.25	3.020 (4)	152
O8—H8*B*⋯O2^ii^	0.85	1.96	2.730 (4)	151

Symmetry codes: (i) 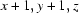; (ii) 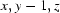.

**Fig. 1.**
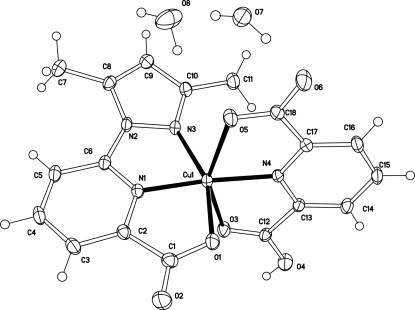
The structure of the title compound (I) showing 50% proability displacement ellipsoids and the atom-numbering scheme.

**Fig. 2.**
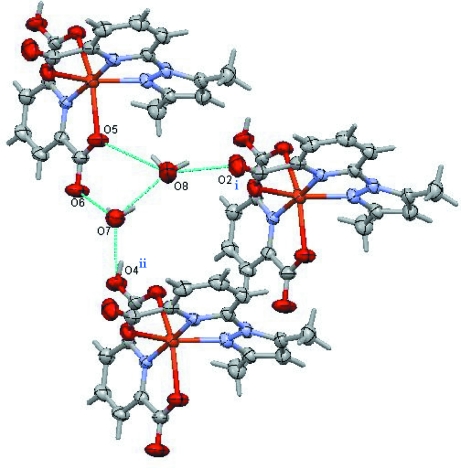
Detail of (I), showing the O—H···O hydrogen bonds. Displacement ellipsoids are drawn at the 50% probability level and the hydrogen bonds are indiacted by dashed lines. [Symmetry codes:(i) x, -1+y, z; (ii) -1+x, -1+y, z.]

**Fig. 3.**
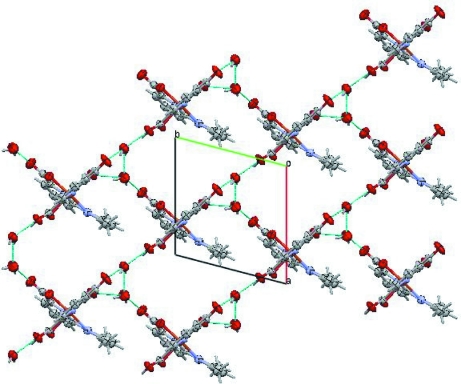
Crystal packing of (I) showing the hydrogen bonding interactions.

## supplementary crystallographic information

### Comment

Recently we reported the crystal structures of bis(6-(3,5-dimethyl-1*H*-pyrazol-1-yl)picolinato)zinc(II) trihydrate [Yin *et al.*, 2007] and bis[3-chloro-6-(3,5-dimethyl-1*H*-pyrazol-1-yl)picolinato]cobalt(II) hemipentahydrate [Zhao *et al.*, 2007]. As a continuation of these investigations, we report here the crystal structure of a new copper(II) complex with the ligand 3-chloro-6-(3,5-dimethyl-1 *H*-pyrazol-1-yl) picolinic acid (CDPA) and pyridine-2,6-dicarboxylate (PDBL). (Fig.1).

The title compound, (I), consists of a central asymmetric copper(II) complex cation and two uncoordinated water molecules. The compound is isostructural with its Ni derivative [Feng *et al.*, 2008]. In the cation (Fig.1), the Cu atom is six-coordinated by three N atoms and three O atoms from CDPA and PDBL ligands. The three diagonal angles for the Cu(II) octahedron range from 150.7 (4)° to 173.7 (2)°, the dihedral angle between the planes of the two ligands is 80.5 (6)°, which indicates a slightly distorted octahedral geometry around the Cu^II^atom.

Analysis of the crystal packing of the title compound reveals the existence of multiple intermolecular O—H···O hydrogen bonds between the mononuclear subunits and the lattice water molecules (Figure 2), forming a two-dimensional hydrogen-bonded sheet perpendicular to the c-axis of the structure (Figure 3). In this sheet the two interstitial water molecules are connecting three complex molecules with each other: The protonated carboxyl oxygen atom O4 acts as a hydrogen donor towards O7 of one of the water molecules. One of the H atoms of O7 in turn binds with the second water molecule, and each one H atom of both water molecules acts as a hydrogen bonding donor towards the two carboxyl O atoms O5 and O6 of a neighboring complex. The last remaining water H atom (H8b) makes the connection to the third complex connected by the two water molecules (Figure 2). The carboxylate group that acts as an H bond acceptor towards both water molecules via both of its O atoms O5 and O6 exhibits a delocalized π system with nearly identical C—O distances.

### Experimental

3-Chloro-(6-(3,5-dimethyl-1*H*-pyrazol-1-yl))picolinic acid, pyridine-2,6-dicarboxylic acid and CuCl_2_.6H_2_O were available commercially and were used without further purification. Equimolar amounts of 3-chloro-6-(3,5-dimethyl-1*H*-pyrazol-1-yl) picolinic acid (0.5 mmol, 125 mg) and pyridine-2,6-dicarboxylic acid (0.5 mmol, 83 mg) were dissolved in anhydrous alcohol (15 ml). The mixture was stirred to give a clear solution, to this solution was added CuCl_2_.6H_2_O (0.5 mmol, 113 mg) in anhydrous alcohol (10 ml). After keeping the resulting solution in air to evaporate about half of the solvents, blue prisms of the title compound were formed. The crystals were isolated, washed with alcohol three times and dried in a vacuum desiccator using silica gel (Yield 75%). Elemental analysis: found: C, 44.85; H, 3.78; N, 11.65%. calc. for C_18_H_18_CuN_4_O_8_: C, 44.86; H, 3.76; N, 11.63%.

### Refinement

H atoms on C atoms were positioned geometrically and refined using a riding model with C–H = 0.93–0.96 Å and *U*_iso_(H) = 1.2–1.5*U*_eq_(C). The water H atoms were located in difference density Fourier maps and refined using a riding model with O–H = 0.82 Å and *U*_iso_(H) = 1.5*U*_eq_(O).

### Crystal data

**Table:**

[Cu(C_7_H_4_NO_4_)(C_11_H_10_N_3_O_2_)]·2H_2_O	*Z* = 2
*M**_r_* = 481.90	*F*_000_ = 494
Triclinic, *P*1	*D*_x_ = 1.653 Mg m^−^^3^
*a* = 9.0040 (10) Å	Mo *K*α radiation λ = 0.71073 Å
*b* = 9.0360 (10) Å	Cell parameters from 3268 reflections
*c* = 12.6760 (15) Å	θ = 2.3–27.8º
α = 103.932 (3)º	µ = 1.19 mm^−^^1^
β = 90.289 (2)º	*T* = 298 (2) K
γ = 104.177 (3)º	Block, blue
*V* = 968.25 (19) Å^3^	0.39 × 0.35 × 0.32 mm

### Data collection

**Table:**

Bruker SMART CCD area-detector diffractometer	3361 independent reflections
Radiation source: fine-focus sealed tube	2844 reflections with *I* > 2σ(*I*)
Monochromator: graphite	*R*_int_ = 0.014
*T* = 298(2) K	θ_max_ = 25.0º
φ and ω scans	θ_min_ = 1.7º
Absorption correction: multi-scan(SADABS; Sheldrick, 1996)	*h* = −10→6
*T*_min_ = 0.655, *T*_max_ = 0.684	*k* = −10→10
5068 measured reflections	*l* = −14→15

### Refinement

**Table:**

Refinement on *F*^2^	Secondary atom site location: difference Fourier map
Least-squares matrix: full	Hydrogen site location: inferred from neighbouring sites
*R*[*F*^2^ > 2σ(*F*^2^)] = 0.043	H-atom parameters constrained
*wR*(*F*^2^) = 0.113	*w* = 1/[σ^2^(*F*_o_^2^) + (0.0647*P*)^2^ + 0.7713*P*] where *P* = (*F*_o_^2^ + 2*F*_c_^2^)/3
*S* = 1.03	(Δ/σ)_max_ < 0.001
3361 reflections	Δρ_max_ = 0.39 e Å^−^^3^
280 parameters	Δρ_min_ = −0.30 e Å^−^^3^
Primary atom site location: structure-invariant direct methods	Extinction correction: none

### Special details

**Table:**

Geometry. All e.s.d.'s (except the e.s.d. in the dihedral angle between two l.s. planes) are estimated using the full covariance matrix. The cell e.s.d.'s are taken into account individually in the estimation of e.s.d.'s in distances, angles and torsion angles; correlations between e.s.d.'s in cell parameters are only used when they are defined by crystal symmetry. An approximate (isotropic) treatment of cell e.s.d.'s is used for estimating e.s.d.'s involving l.s. planes.
Refinement. Refinement of *F*^2^ against ALL reflections. The weighted *R*-factor *wR* and goodness of fit *S* are based on *F*^2^, conventional *R*-factors *R* are based on *F*, with *F* set to zero for negative *F*^2^. The threshold expression of *F*^2^ > σ(*F*^2^) is used only for calculating *R*-factors(gt) *etc*. and is not relevant to the choice of reflections for refinement. *R*-factors based on *F*^2^ are statistically about twice as large as those based on *F*, and *R*- factors based on ALL data will be even larger.

### Fractional atomic coordinates and isotropic or equivalent isotropic displacement parameters (Å^2^)

**Table:**

	*x*	*y*	*z*	*U*_iso_*/*U*_eq_	
Cu1	0.72104 (5)	0.95250 (5)	0.26981 (3)	0.03369 (16)	
N1	0.7309 (3)	0.9975 (3)	0.4271 (2)	0.0318 (6)	
N2	0.8342 (3)	0.7887 (3)	0.4100 (2)	0.0358 (6)	
N3	0.8359 (3)	0.7890 (3)	0.3006 (2)	0.0350 (6)	
N4	0.6970 (3)	0.9233 (3)	0.1106 (2)	0.0296 (6)	
O1	0.6153 (3)	1.1315 (3)	0.3022 (2)	0.0476 (6)	
O2	0.5465 (4)	1.2990 (4)	0.4420 (2)	0.0662 (8)	
O3	0.9435 (3)	1.1261 (3)	0.2279 (2)	0.0472 (7)	
O4	1.0063 (3)	1.2352 (3)	0.0888 (2)	0.0578 (8)	
H4	1.0757	1.2945	0.1323	0.087*	
O5	0.5089 (3)	0.7472 (3)	0.21665 (19)	0.0498 (7)	
O6	0.3550 (4)	0.6217 (4)	0.0688 (2)	0.0735 (10)	
O7	0.2133 (3)	0.4600 (3)	0.1912 (2)	0.0576 (7)	
H7D	0.2617	0.4484	0.2451	0.069*	
H7E	0.2754	0.4928	0.1467	0.069*	
O8	0.4582 (4)	0.4587 (4)	0.3057 (3)	0.0964 (13)	
H8A	0.5002	0.5498	0.2972	0.116*	
H8B	0.5112	0.4395	0.3540	0.116*	
C1	0.6077 (4)	1.1920 (4)	0.4025 (3)	0.0419 (8)	
C2	0.6808 (4)	1.1214 (4)	0.4800 (3)	0.0359 (8)	
C3	0.6976 (4)	1.1722 (4)	0.5913 (3)	0.0459 (9)	
H3	0.6632	1.2585	0.6279	0.055*	
C4	0.7675 (4)	1.0906 (5)	0.6474 (3)	0.0463 (9)	
H4A	0.7813	1.1239	0.7230	0.056*	
C5	0.8171 (4)	0.9614 (4)	0.5940 (3)	0.0427 (9)	
H5	0.8637	0.9066	0.6318	0.051*	
C6	0.7943 (4)	0.9166 (4)	0.4808 (3)	0.0337 (7)	
C7	0.8598 (6)	0.6154 (5)	0.5339 (3)	0.0578 (11)	
H7A	0.8610	0.5071	0.5244	0.087*	
H7B	0.7682	0.6328	0.5675	0.087*	
H7C	0.9481	0.6821	0.5795	0.087*	
C8	0.8633 (4)	0.6533 (4)	0.4257 (3)	0.0402 (8)	
C9	0.8869 (5)	0.5692 (5)	0.3260 (3)	0.0474 (9)	
H9	0.9105	0.4719	0.3103	0.057*	
C10	0.8691 (4)	0.6564 (4)	0.2504 (3)	0.0394 (8)	
C11	0.8836 (5)	0.6158 (5)	0.1306 (3)	0.0558 (11)	
H11A	0.8756	0.7030	0.1020	0.084*	
H11B	0.8031	0.5247	0.0967	0.084*	
H11C	0.9814	0.5936	0.1158	0.084*	
C12	0.9239 (4)	1.1361 (4)	0.1343 (3)	0.0354 (8)	
C13	0.7922 (4)	1.0210 (4)	0.0612 (3)	0.0325 (7)	
C14	0.7664 (4)	1.0143 (4)	−0.0471 (3)	0.0418 (8)	
H14	0.8322	1.0841	−0.0797	0.050*	
C15	0.6420 (5)	0.9030 (5)	−0.1072 (3)	0.0460 (9)	
H15	0.6246	0.8957	−0.1809	0.055*	
C16	0.5451 (4)	0.8038 (4)	−0.0566 (3)	0.0437 (9)	
H16	0.4605	0.7286	−0.0953	0.052*	
C17	0.5747 (4)	0.8171 (4)	0.0524 (3)	0.0350 (8)	
C18	0.4718 (4)	0.7214 (4)	0.1191 (3)	0.0404 (8)	

### Atomic displacement parameters (Å^2^)

**Table:**

	*U*^11^	*U*^22^	*U*^33^	*U*^12^	*U*^13^	*U*^23^
Cu1	0.0395 (3)	0.0366 (2)	0.0240 (2)	0.00751 (18)	−0.00296 (16)	0.00805 (16)
N1	0.0295 (15)	0.0370 (15)	0.0271 (14)	0.0050 (12)	−0.0005 (11)	0.0082 (11)
N2	0.0410 (16)	0.0431 (16)	0.0251 (14)	0.0105 (13)	−0.0025 (12)	0.0124 (12)
N3	0.0421 (17)	0.0390 (15)	0.0244 (14)	0.0096 (13)	−0.0014 (12)	0.0099 (12)
N4	0.0303 (14)	0.0297 (13)	0.0268 (14)	0.0040 (11)	−0.0029 (11)	0.0072 (11)
O1	0.0601 (17)	0.0491 (15)	0.0353 (14)	0.0208 (13)	−0.0082 (12)	0.0070 (11)
O2	0.083 (2)	0.0616 (18)	0.0598 (19)	0.0395 (17)	−0.0055 (16)	0.0045 (15)
O3	0.0454 (15)	0.0490 (15)	0.0385 (15)	−0.0078 (12)	−0.0113 (12)	0.0148 (11)
O4	0.0518 (17)	0.0564 (16)	0.0536 (17)	−0.0187 (14)	−0.0063 (13)	0.0252 (13)
O5	0.0479 (15)	0.0552 (15)	0.0326 (14)	−0.0134 (13)	−0.0022 (11)	0.0122 (11)
O6	0.066 (2)	0.077 (2)	0.0546 (18)	−0.0361 (17)	−0.0245 (15)	0.0292 (16)
O7	0.0581 (18)	0.0593 (17)	0.0539 (17)	0.0052 (14)	−0.0020 (14)	0.0211 (14)
O8	0.076 (2)	0.074 (2)	0.147 (4)	0.0085 (19)	−0.033 (2)	0.055 (2)
C1	0.040 (2)	0.0373 (19)	0.045 (2)	0.0083 (16)	−0.0040 (16)	0.0062 (16)
C2	0.0344 (19)	0.0364 (18)	0.0319 (18)	0.0025 (15)	0.0008 (14)	0.0058 (14)
C3	0.053 (2)	0.044 (2)	0.0331 (19)	0.0061 (18)	0.0057 (17)	0.0002 (16)
C4	0.050 (2)	0.057 (2)	0.0234 (17)	0.0041 (19)	0.0008 (16)	0.0042 (16)
C5	0.045 (2)	0.053 (2)	0.0295 (18)	0.0078 (18)	−0.0021 (16)	0.0146 (16)
C6	0.0324 (18)	0.0396 (18)	0.0292 (17)	0.0057 (15)	−0.0008 (14)	0.0122 (14)
C7	0.079 (3)	0.057 (2)	0.045 (2)	0.021 (2)	−0.001 (2)	0.0243 (19)
C8	0.039 (2)	0.044 (2)	0.041 (2)	0.0103 (16)	−0.0043 (16)	0.0179 (16)
C9	0.058 (3)	0.043 (2)	0.044 (2)	0.0169 (18)	−0.0040 (18)	0.0120 (17)
C10	0.040 (2)	0.045 (2)	0.0324 (18)	0.0115 (16)	−0.0030 (15)	0.0074 (15)
C11	0.075 (3)	0.059 (2)	0.036 (2)	0.025 (2)	0.002 (2)	0.0070 (18)
C12	0.0322 (18)	0.0374 (18)	0.0367 (19)	0.0032 (15)	−0.0038 (15)	0.0151 (15)
C13	0.0334 (18)	0.0354 (17)	0.0299 (17)	0.0070 (14)	0.0002 (14)	0.0121 (14)
C14	0.045 (2)	0.048 (2)	0.0338 (19)	0.0052 (17)	0.0028 (16)	0.0183 (16)
C15	0.054 (2)	0.057 (2)	0.0253 (17)	0.0098 (19)	−0.0060 (16)	0.0118 (16)
C16	0.047 (2)	0.048 (2)	0.0294 (18)	0.0035 (17)	−0.0093 (16)	0.0054 (15)
C17	0.041 (2)	0.0312 (17)	0.0300 (17)	0.0064 (15)	−0.0037 (15)	0.0052 (14)
C18	0.040 (2)	0.0379 (18)	0.037 (2)	−0.0032 (16)	−0.0065 (16)	0.0101 (15)

### Geometric parameters (Å, °)

**Table:**

Cu1—N1	1.934 (3)	C3—C4	1.387 (5)
Cu1—N4	1.975 (3)	C3—H3	0.9300
Cu1—O1	2.032 (3)	C4—C5	1.377 (5)
Cu1—N3	2.104 (3)	C4—H4A	0.9300
Cu1—O5	2.279 (2)	C5—C6	1.394 (5)
Cu1—O3	2.372 (2)	C5—H5	0.9300
N1—C6	1.327 (4)	C7—C8	1.490 (5)
N1—C2	1.340 (4)	C7—H7A	0.9600
N2—C8	1.370 (4)	C7—H7B	0.9600
N2—N3	1.388 (4)	C7—H7C	0.9600
N2—C6	1.406 (4)	C8—C9	1.357 (5)
N3—C10	1.318 (4)	C9—C10	1.410 (5)
N4—C13	1.344 (4)	C9—H9	0.9300
N4—C17	1.345 (4)	C10—C11	1.489 (5)
O1—C1	1.266 (4)	C11—H11A	0.9600
O2—C1	1.233 (4)	C11—H11B	0.9600
O3—C12	1.226 (4)	C11—H11C	0.9600
O4—C12	1.269 (4)	C12—C13	1.505 (5)
O4—H4	0.8200	C13—C14	1.376 (5)
O5—C18	1.230 (4)	C14—C15	1.385 (5)
O6—C18	1.256 (4)	C14—H14	0.9300
O7—H7D	0.8498	C15—C16	1.369 (5)
O7—H7E	0.8498	C15—H15	0.9300
O8—H8A	0.8500	C16—C17	1.377 (5)
O8—H8B	0.8500	C16—H16	0.9300
C1—C2	1.519 (5)	C17—C18	1.512 (5)
C2—C3	1.370 (5)		
			
N1—Cu1—N4	173.70 (11)	C6—C5—H5	121.5
N1—Cu1—O1	80.55 (11)	N1—C6—C5	121.3 (3)
N4—Cu1—O1	93.19 (10)	N1—C6—N2	112.0 (3)
N1—Cu1—N3	77.82 (11)	C5—C6—N2	126.8 (3)
N4—Cu1—N3	108.45 (10)	C8—C7—H7A	109.5
O1—Cu1—N3	158.36 (10)	C8—C7—H7B	109.5
N1—Cu1—O5	103.92 (10)	H7A—C7—H7B	109.5
N4—Cu1—O5	76.16 (10)	C8—C7—H7C	109.5
O1—Cu1—O5	98.55 (11)	H7A—C7—H7C	109.5
N3—Cu1—O5	87.33 (11)	H7B—C7—H7C	109.5
N1—Cu1—O3	105.16 (10)	C9—C8—N2	106.0 (3)
N4—Cu1—O3	75.33 (9)	C9—C8—C7	130.9 (3)
O1—Cu1—O3	89.65 (11)	N2—C8—C7	123.1 (3)
N3—Cu1—O3	95.34 (10)	C8—C9—C10	107.4 (3)
O5—Cu1—O3	150.70 (9)	C8—C9—H9	126.3
C6—N1—C2	121.3 (3)	C10—C9—H9	126.3
C6—N1—Cu1	121.7 (2)	N3—C10—C9	110.2 (3)
C2—N1—Cu1	116.9 (2)	N3—C10—C11	121.2 (3)
C8—N2—N3	110.9 (3)	C9—C10—C11	128.5 (3)
C8—N2—C6	132.6 (3)	C10—C11—H11A	109.5
N3—N2—C6	116.4 (3)	C10—C11—H11B	109.5
C10—N3—N2	105.5 (3)	H11A—C11—H11B	109.5
C10—N3—Cu1	140.2 (2)	C10—C11—H11C	109.5
N2—N3—Cu1	110.3 (2)	H11A—C11—H11C	109.5
C13—N4—C17	119.1 (3)	H11B—C11—H11C	109.5
C13—N4—Cu1	120.4 (2)	O3—C12—O4	126.6 (3)
C17—N4—Cu1	120.1 (2)	O3—C12—C13	119.5 (3)
C1—O1—Cu1	114.9 (2)	O4—C12—C13	113.9 (3)
C12—O3—Cu1	108.3 (2)	N4—C13—C14	121.2 (3)
C12—O4—H4	109.5	N4—C13—C12	114.5 (3)
C18—O5—Cu1	111.8 (2)	C14—C13—C12	124.3 (3)
H7D—O7—H7E	110.6	C13—C14—C15	119.6 (3)
H8A—O8—H8B	109.0	C13—C14—H14	120.2
O2—C1—O1	126.8 (4)	C15—C14—H14	120.2
O2—C1—C2	118.0 (3)	C16—C15—C14	119.0 (3)
O1—C1—C2	115.1 (3)	C16—C15—H15	120.5
N1—C2—C3	121.1 (3)	C14—C15—H15	120.5
N1—C2—C1	112.1 (3)	C15—C16—C17	119.0 (3)
C3—C2—C1	126.7 (3)	C15—C16—H16	120.5
C2—C3—C4	117.7 (3)	C17—C16—H16	120.5
C2—C3—H3	121.1	N4—C17—C16	122.1 (3)
C4—C3—H3	121.1	N4—C17—C18	113.7 (3)
C5—C4—C3	121.6 (3)	C16—C17—C18	124.2 (3)
C5—C4—H4A	119.2	O5—C18—O6	125.8 (3)
C3—C4—H4A	119.2	O5—C18—C17	117.9 (3)
C4—C5—C6	116.9 (3)	O6—C18—C17	116.2 (3)
C4—C5—H5	121.5		
			
O1—Cu1—N1—C6	179.7 (3)	O1—C1—C2—C3	175.1 (3)
N3—Cu1—N1—C6	−1.0 (3)	N1—C2—C3—C4	−0.3 (5)
O5—Cu1—N1—C6	83.1 (3)	C1—C2—C3—C4	179.8 (3)
O3—Cu1—N1—C6	−93.3 (3)	C2—C3—C4—C5	−0.8 (6)
O1—Cu1—N1—C2	−5.0 (2)	C3—C4—C5—C6	0.2 (5)
N3—Cu1—N1—C2	174.3 (3)	C2—N1—C6—C5	−2.7 (5)
O5—Cu1—N1—C2	−101.6 (2)	Cu1—N1—C6—C5	172.4 (2)
O3—Cu1—N1—C2	82.0 (2)	C2—N1—C6—N2	178.1 (3)
C8—N2—N3—C10	−1.3 (4)	Cu1—N1—C6—N2	−6.8 (4)
C6—N2—N3—C10	−177.3 (3)	C4—C5—C6—N1	1.5 (5)
C8—N2—N3—Cu1	160.9 (2)	C4—C5—C6—N2	−179.4 (3)
C6—N2—N3—Cu1	−15.0 (3)	C8—N2—C6—N1	−160.3 (3)
N1—Cu1—N3—C10	161.2 (4)	N3—N2—C6—N1	14.5 (4)
N4—Cu1—N3—C10	−18.1 (4)	C8—N2—C6—C5	20.6 (6)
O1—Cu1—N3—C10	163.0 (3)	N3—N2—C6—C5	−164.6 (3)
O5—Cu1—N3—C10	56.4 (4)	N3—N2—C8—C9	1.4 (4)
O3—Cu1—N3—C10	−94.4 (4)	C6—N2—C8—C9	176.4 (4)
N1—Cu1—N3—N2	8.5 (2)	N3—N2—C8—C7	−176.3 (3)
N4—Cu1—N3—N2	−170.80 (19)	C6—N2—C8—C7	−1.3 (6)
O1—Cu1—N3—N2	10.3 (4)	N2—C8—C9—C10	−0.8 (4)
O5—Cu1—N3—N2	−96.3 (2)	C7—C8—C9—C10	176.6 (4)
O3—Cu1—N3—N2	112.9 (2)	N2—N3—C10—C9	0.8 (4)
O1—Cu1—N4—C13	79.0 (3)	Cu1—N3—C10—C9	−152.7 (3)
N3—Cu1—N4—C13	−100.6 (3)	N2—N3—C10—C11	−179.0 (3)
O5—Cu1—N4—C13	177.0 (3)	Cu1—N3—C10—C11	27.5 (6)
O3—Cu1—N4—C13	−9.8 (2)	C8—C9—C10—N3	0.0 (4)
O1—Cu1—N4—C17	−93.3 (2)	C8—C9—C10—C11	179.8 (4)
N3—Cu1—N4—C17	87.1 (3)	Cu1—O3—C12—O4	168.4 (3)
O5—Cu1—N4—C17	4.7 (2)	Cu1—O3—C12—C13	−12.6 (4)
O3—Cu1—N4—C17	177.9 (3)	C17—N4—C13—C14	0.1 (5)
N1—Cu1—O1—C1	2.1 (3)	Cu1—N4—C13—C14	−172.3 (3)
N4—Cu1—O1—C1	−178.6 (3)	C17—N4—C13—C12	179.3 (3)
N3—Cu1—O1—C1	0.4 (5)	Cu1—N4—C13—C12	6.9 (4)
O5—Cu1—O1—C1	104.9 (3)	O3—C12—C13—N4	5.8 (5)
O3—Cu1—O1—C1	−103.3 (3)	O4—C12—C13—N4	−175.1 (3)
N1—Cu1—O3—C12	−161.4 (2)	O3—C12—C13—C14	−175.0 (3)
N4—Cu1—O3—C12	12.1 (2)	O4—C12—C13—C14	4.1 (5)
O1—Cu1—O3—C12	−81.3 (2)	N4—C13—C14—C15	−1.3 (5)
N3—Cu1—O3—C12	119.8 (2)	C12—C13—C14—C15	179.6 (3)
O5—Cu1—O3—C12	25.8 (4)	C13—C14—C15—C16	1.4 (6)
N1—Cu1—O5—C18	168.6 (3)	C14—C15—C16—C17	−0.4 (6)
N4—Cu1—O5—C18	−4.9 (3)	C13—N4—C17—C16	0.9 (5)
O1—Cu1—O5—C18	86.3 (3)	Cu1—N4—C17—C16	173.3 (3)
N3—Cu1—O5—C18	−114.6 (3)	C13—N4—C17—C18	−176.4 (3)
O3—Cu1—O5—C18	−18.5 (4)	Cu1—N4—C17—C18	−4.0 (4)
Cu1—O1—C1—O2	−178.3 (3)	C15—C16—C17—N4	−0.8 (6)
Cu1—O1—C1—C2	0.8 (4)	C15—C16—C17—C18	176.3 (4)
C6—N1—C2—C3	2.0 (5)	Cu1—O5—C18—O6	−175.8 (4)
Cu1—N1—C2—C3	−173.3 (3)	Cu1—O5—C18—C17	4.4 (4)
C6—N1—C2—C1	−178.0 (3)	N4—C17—C18—O5	−0.8 (5)
Cu1—N1—C2—C1	6.7 (4)	C16—C17—C18—O5	−178.1 (4)
O2—C1—C2—N1	174.4 (3)	N4—C17—C18—O6	179.3 (3)
O1—C1—C2—N1	−4.8 (4)	C16—C17—C18—O6	2.0 (6)
O2—C1—C2—C3	−5.6 (6)		

### Hydrogen-bond geometry (Å, °)

**Table:**

*D*—H···*A*	*D*—H	H···*A*	*D*···*A*	*D*—H···*A*
O4—H4···O7^i^	0.82	1.69	2.477 (4)	159
O7—H7D···O8	0.85	1.89	2.637 (4)	145
O7—H7E···O6	0.85	1.73	2.524 (4)	154
O8—H8A···O5	0.85	2.25	3.020 (4)	152
O8—H8B···O2^ii^	0.85	1.96	2.730 (4)	151

Symmetry codes: (i) *x*+1, *y*+1, *z*; (ii) *x*, *y*−1, *z*.
